# Hot Weather and Violence Against Women: A Global Scoping Review

**DOI:** 10.3390/ijerph22071069

**Published:** 2025-07-03

**Authors:** Chiratidzo Hope Mulambo, Rishu Thakur, Supriya Mathew

**Affiliations:** 1 Charles Darwin University, Casuarina, NT 0810, Australia; chiratidzo.mulambo@students.cdu.edu.au; 2 Remote Health Systems and Climate Change Centre, Menzies School of Health Research, Charles Darwin University, Alice Springs, NT 0870, Australia; rishu.thakur@menzies.edu.au

**Keywords:** climate change, heatwaves, high temperatures, gender-based violence, intimate partner violence, public health, socio-environmental stressors

## Abstract

Temperature increases due to climatic changes have been increasingly recognized as posing significant public health challenges, with wide-ranging socio-economic implications. This scoping review examines the relationship between high temperatures and violence against women (VAW) globally. Nine studies from both high-income and low- and middle-income countries were included in this review. The findings suggest an overall positive association between high temperatures and rates of VAW. Theoretical frameworks, including the temperature–aggression hypothesis and routine activity theory, offer insights into the mechanisms driving this relationship. Key risk factors such as socioeconomic status, urban heat island effects, rurality, patriarchal norms, and alcohol consumption were considered to be risk factors affecting rates of VAW. Despite growing evidence, research gaps persist, particularly in regions with high rates of VAW and in the form of qualitative studies that capture women’s lived experiences. The positive associations between temperature and VAW underscore the urgency of integrating gender-sensitive strategies into climate adaptation policies to mitigate the compounding risks of climate change and gender-based violence.

## 1. Introduction

The Intergovernmental Panel on Climate Change (IPCC) Sixth Assessment Report projections indicate an increase in global temperatures of 3.2 °C or more by 2100 under a high-emission scenario [1]. These projections are of concern, given high temperatures have already been linked to increased mortality, morbidity, and health service usage, particularly for mental health issues, cardiovascular and respiratory diseases, and occupational injuries [2]. High temperatures have also been observed to affect social behavior, with studies showing increased aggression, crime, and violence in hot weather [3,4]. According to World Health Organisation (WHO), one in three women worldwide has experienced physical or sexual violence, often by an intimate partner [5]. Increasing temperatures exacerbate the risk of violence against women (VAW), intensifying pre-existing gender-specific vulnerabilities.

Two theoretical frameworks provide insight into the relationship between temperature and violence. The temperature–aggression hypothesis posits that heat-induced physiological stress, such as increased irritation and reduced self-control, raises the likelihood of aggressive behaviors [6]. Routine activity theory suggests that environmental changes, such as warmer climates, alter social interactions and routines, increasing the potential for violence [7]. Thus, the association between heat and crime is determined by a variety of processes, including social (that is, people may spend more time outdoors or indoors) [8] and physiological factors (dehydration, exhaustion and discomfort which heightens irritation) [9].

A global study estimated that each 1 °C increase in annual temperature is associated with an average 6% rise in homicide rates [8]. As global temperatures rise and heatwaves become more severe [1], understanding and addressing the complex interplay between environmental stressors such as extreme heat events and social inequities will be critical for building resilience and ensuring equitable outcomes. It is concerning that the effects of climate change on violence vary regionally, with the most pronounced impacts occurring in regions already burdened by high homicide rates and existing social instability [8].

There have been reviews published linking extreme climate events to VAW [10] and high temperatures to crime [11], but there have not been any reviews that specifically focus on the effects of high temperatures on VAW. This scoping review aims to synthesize literature exploring the relationship between high temperatures and VAW globally. The scoping review explores the following research questions:

- What are the main trends reported for high temperatures and VAW?
- Are there evidence gaps that need to be explored further under a changing climate?
- What types of violence (for example, physical, emotional, sexual) are most reported in connection with hot weather events?
- What are the sources of primary and secondary data used in the studies?
- How do socioeconomic status, literacy rates, climate characteristics, and cultural factors mediate VAW?

## 2. Materials and Methods

### 2.1. Search Strategy and Databases

The scoping review followed Joanna Briggs Institute’s (JBI) guidelines for scoping reviews [12]. A search strategy was drafted to identify relevant studies related to hot weather and VAW. Databases such as Web of Science, PubMed, Scopus and Google Scholar were searched using a combination of keywords related to hot weather and VAW. The reference list of all included sources of evidence was then screened for additional studies.

### 2.2. Search Terms

Databases were searched using a combination of search terms related to hot weather, violence and women. A search string was developed with assistance from a librarian at Charles Darwin University as follows: (“hot weather” OR “high temperature” OR heat OR “extreme temperature” OR “climate change”) AND (violence OR aggression OR “domestic violence” OR “intimate partner violence” OR “sexual violence” OR “gender-based violence”) AND (women OR gender OR female).

### 2.3. Source of Evidence Selection and Data Extraction

Title and abstract screening were performed by two independent reviewers for assessment against the inclusion criteria for the review, followed by full-text screening of selected citations. The Preferred Reporting Items for Systematic Reviews and Meta-Analyses extension for Scoping Reviews (PRISMA-ScR) guided the selection process and the reporting of results [13]. Data was extracted by two reviewers. The data extraction table included specific details about the source references, country of study, population characteristics, temperature context, climate zone, design/methodology, groups at increased risk, and key findings relevant to the review questions.

## 3. Results

### 3.1. Study Characteristics

**Figure 1:**
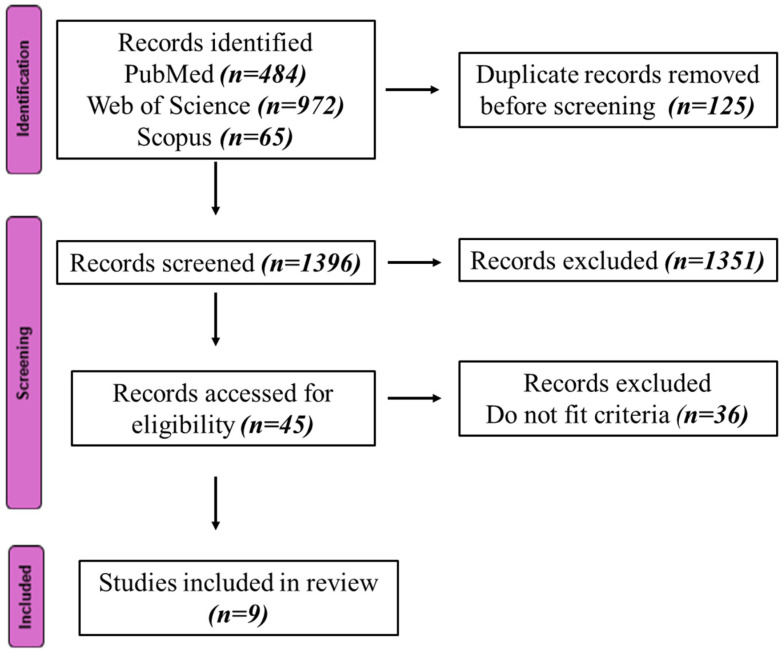
PRISMA flowchart.

One thousand five hundred and twenty-one studies were identified from the three databases. One thousand three hundred and fifty-one articles were excluded during the first screening. Of the forty-five possibly eligible studies assessed for full-text eligibility, thirty-six articles were excluded. The main reasons for exclusion included articles that did not specifically report results on violence or crime against women, studies focusing solely on seasonal variations in crime or violence, non-peer-reviewed publications, and review articles or commentaries lacking primary or secondary data analysis. Articles that focused solely on droughts, without explicitly addressing extreme temperatures as an exposure variable, were excluded. Nine articles were finally included in this scoping review. The PRISMA flow diagram in Figure 1 summarizes the selection process. The studies were carried out in both High-Income Countries (HICs), such as the United States and Spain, and Low- and Middle-Income Countries (LMICs), such as India, Nepal, and several African countries. Studies included data from the South Asian region [Bangladesh, India, Nepal, and Pakistan] [14,15], several countries in Africa [15], Australia [16], Spain [17], Russia [18], United States [19,20,21,22], and (see Table 1).

### 3.2. Data Sources and Study Designs

All nine studies used quantitative methodologies (see Table 2). The study examined diverse outcome variables to capture the heat-related impacts on VAW. Data on VAW included crime police call records [19,20], crimes recorded by the police and/or the Department of Public Safety [16,21] and demographic and health survey data [14,15]. Different temperature exposure variables were also used in the studies. It included average daily temperatures [14,18,19,20,21,22], monthly average temperatures [20], hourly temperatures [20], annual average temperatures [15], heatwaves [defined as when the daily maximum temperatures surpass a threshold of 34 °C] [17], and daily maximum temperatures [16].

### 3.3. Association Between Hot Weather and Violence Against Women

A study focused on three South Asian countries: India, Nepal, and Pakistan, found that Intimate Partner Violence (IPV) increased with temperature [14]. IPV was estimated to increase by 4.5% for every 1% rise in the mean annual temperature, with India recording the highest rates of IPV. The study projected a 21% increase in IPV by the end of the 21st century under an unlimited emission scenario [14]. In the United States, it was found that incidents of VAW, specifically domestic violence, were influenced by temporal variables such as time of day and ambient temperature [19]. This study also showed that domestic violence was linked with other factors, such as alcohol consumption, which can increase during hot weather periods. In the Minneapolis study [20], it was found that domestic violence rose on days when the temperature peaked over 27 °C, indicating a correlation between rising temperatures and an increase in hostility. During heatwave periods in Madrid, a significant increase in hotline calls and IPV incidents was recorded [17]. In Madrid, the incidence of IPV rose one day after a heatwave began, with the risk of intimate partner femicides peaking three days after the heatwave’s onset [17]. In Russia, extreme temperatures were found to increase violent mortality, but the effects were unequal across gender and age groups [18]. This study also found increased violent mortality during the weekends, where females were more victimized than men. In a study that included survey data from 34 developing countries, a positive association between increased temperatures and IPV was found [15]. The study found that a rise in mean annual temperature by 5.9 °C increased the probability of IPV by 3.7% across countries.

#### Association Between Hot Weather and Different Types of VAW

Several studies reported different types of VAW. The types of violence reported included physical, sexual, and emotional violence. Physical abuse also included slapping, punching, and pushing. Nguyen (2024) reported that in developing countries a one standard deviation increase in temperature (~6 °C) in the women’s location in the past 12 months was associated with an increase in Less Severe Violence (14.7%), More Severe Violence (9.3%), Emotional Abuse (8.2%), and Sexual Abuse (6.1%), respectively [15]. In South Asia, physical violence was the most common form (23.0%) of violence against women, followed by emotional violence (12.5%) and sexual violence (9.5%) [14]. This South Asian study also estimated that for every 1 °C rise in temperature, the prevalence of physical violence increased by 6.55%, sexual violence by 6.21%, and emotional violence by 1.39%. Another study conducted in Minneapolis using data from between 1985 and 1988 showed that temporal conditions influenced domestic violence but had less influence on other sexual crimes such as rape [20]. In US, an increase in daily mean temperature of 5 °C was associated with a 4.5% increase in sex offenses over the following 0–8 days, with higher risks of offenses such as sodomy, fondling and rape [21]. In New South Wales (Australia), it was reported that sexual assaults peaked around 30 °C for both inside and outside settings, with 15% of the assaults occurring in indoor settings [16].

### 3.4. Risk Factors Affecting VAW

Studies noted that hot weather and VAW are mediated by several risk factors, including socioeconomic status, literacy, climate, and cultural factors. Women from low-income households were found to be more vulnerable to heat-related violence. For example, Henke et al. [22] reported that women in low-income households were more likely to experience IPV. Higher rates of heat-related IPV were linked to economic dependency and patriarchal norms in the Sub-Saharan African locations, especially in the rural areas [15]. This study also found that women in rural areas, poorer households, with lower education, and those living with less-educated partners, were particularly vulnerable to increased IPV due to higher temperatures [15]. Similarly, IPV rates increased by 5.09% in lower-income households and 3.38% in higher-income households in developing countries, disproportionately affecting women in lower-income households [14]. Urban settings were not immune to heat-related violence. Studies from Spain [17] and the United States. Reference [22] revealed that even in urban areas, factors such as socioeconomic disparities exacerbated women’s vulnerability.

The prevalence of violence was also mediated by the level of education of both women and their partners. Increased IPV prevalence among women with low-educated partners was observed in South Asia [14]. Similarly, another survey-based study, including survey data from 34 developing countries, found that women with less education face higher risks of violence [15]. The studies reported that strong established norms in LMICs made women more susceptible to violence, especially in situations where male dominance was associated with honor and control [14,15]. Otrachshenko (2021) observed that in Russia, alcohol use exacerbated aggression and conflict [18]. Henke and Hsu (2020) on the other hand, tested this hypothesis, but did not find IPV to vary between sober and non-sober offenders [22].

## 4. Discussion

This review provides compelling evidence linking hot weather to increased violence against women. In South Asia, IPV prevalence increased by 4.5% for every 1 °C rise in mean annual temperature [14], with similar increasing trends reported in the United States [20] and Russia [18]. Given that IPCC projections estimate a global temperature rise of 1.5 °C above pre-industrial levels by 2030–2035, with possible increases of over 3 °C by the end of the century [1], this trend is alarming. Heatwaves have become more frequent and intense across all continents [23]. A J-shaped relationship between temperature and violence suggests that while extreme heat increases aggression, cold temperatures do not have the same effect [18]. A reduction in the number of cold days due to global warming may further amplify heat-induced aggression [24]. These findings are crucial for climate and health policymakers as rising temperatures may worsen heat-related violence.

Despite research from high-income countries (HICs) such as the United States [22], Spain [17], Russia [18], and Australia [16], as well as low- and middle-income countries (LMICs) in Sub-Saharan Africa and Asia [14,15], many countries with high rates of VAW, such as Ethiopia, Zimbabwe, and regions, such as South Asia and the Middle East that report high rates of violence against women, remain understudied. Studies in low-income countries (LICs) are particularly relevant as these regions are disproportionately affected by climate change and have higher violence rates [25]. For example, Ethiopia reports that 50–76.5% of women have experienced some form of violence [26], while Zimbabwe has an intimate partner violence (IPV) prevalence of 61.3% [27]. Similarly, India reports IPV rates as high as 61.8% [28].

Another key gap in current research is the lack of qualitative studies capturing the lived experiences of women, which is necessary for the understanding of the socio-cultural and psychological factors that exacerbate VAW during periods of extreme heat. VAW is often underreported due to stigma and fear [29]. Complete reliance on secondary data could thus underestimate the risks and fail to explain the root causes of extreme heat-related violence. Context-specific and culturally sensitive research is needed to fully comprehend the complex relationship between heat and violence [5].

Up to 85% of VAW is perpetrated by current or former partners [30]. Younger women, particularly those aged 15–24, are the most affected [31]. During the COVID-19 lockdowns, increased indoor VAW was reported [32,33]. Studies in Russia and Australia found that extreme heat disproportionately affected violence in enclosed spaces, contradicting the routine activity theory, which attributes outdoor violence to social interactions in public spaces [34]. During heatwaves, people spend more time indoors, exacerbating IPV, similar to patterns observed during COVID-19 lockdowns [16]. Lack of access to air conditioning in houses could intensify temperature-related mental stress, which could amplify domestic violence rates [35].

Studies have found that domestic violence rises on days when temperatures exceed a particular threshold (e.g., 27 °C in Minneapolis), supporting the temperature aggression theory that high temperatures impair emotional regulation [20]. In Russia, IPV rates were significantly higher when temperatures exceeded 25 °C, particularly in areas with high unemployment and alcohol consumption [18]. Such variations emphasize the need for localized studies to understand local temperature thresholds.

Socio-economic disparities further exacerbate the effects of heat-related violence against women, particularly in LMICs. Hot weather often coincides with droughts, floods, and other extreme weather events that reduce economic stability [36]. In South Asia and Sub-Saharan Africa, disruptions to agricultural livelihoods lower household incomes, increase food insecurity and compound household inhabitants’ stress [14]. Male unemployment rates correlate with higher IPV rates, whereas female education and employment was observed to be a protective factor [37]. Although urban areas have seen declines in IPV due to increased economic opportunities for women [38], economic empowerment can also challenge traditional power dynamics, potentially leading to backlash and further violence [39]. Rural women, especially those from low-income households, are at heightened risk of IPV during heatwaves [15]. Factors such as unfinished housing, less-educated spouses, early marriage, and dowry disputes further contribute to violence [40]. These findings align with Bronfenbrenner’s ecological systems theory, which highlights how macro-level factors such as economic disparity and climate change shape individual experiences of violence [41].

Cultural and geographical differences also influence how heat-related violence manifests. In South Asia and Sub-Saharan Africa, entrenched patriarchal norms normalize violence against women, with dowry-related violence remaining a significant issue [37]. In Western countries, IPV is more closely tied to economic inequalities, such as gendered pay disparities [22]. Alcohol consumption is also a significant risk factor, with studies showing that alcohol use increases in hotter climates [42].

## 5. Limitations, Future Research, and Implications for Policy and Practice

This review covers studies from both HICs and LMICs, demonstrating that heat-related VAW is a global phenomenon requiring policy attention. A meta-analysis was not conducted as different temperature exposure variables and outcome variables were used in the studies. The different methods used to measure heat-related VAW also hindered cross-study comparisons. Regional and cultural variations in reporting also affect data comparability. Seasonal variations in violence against women were not examined in this review. A key limitation of the review is the absence of qualitative studies, which limits our understanding of how heat influences violence beyond statistical correlations. Future research should focus on qualitative studies to provide deeper insights into women’s lived experiences and resilience strategies. Longitudinal research is necessary to examine how climate change affects violence over time. Studies should also cover underrepresented regions, including the Middle East, South America, and Pacific Island countries.

This review highlights the need for targeted interventions addressing the intersection of climate change and violence against women. Policymakers must integrate gender-sensitive strategies into climate adaptation plans. Providing safe cool shelters (e.g., public cool spaces, green spaces, climate-friendly building designs) and social support during hot weather periods can mitigate the effects of extreme heat on VAW [43]. Social support systems should give special attention to vulnerable women from rural/remote and low-income settings. Economic empowerment programs promoting female employment have been observed to be a mitigating factor [44] and require specific attention. Predictive models linking heat and IPV are required to guide health services, law enforcement, and social agencies to deploy resources appropriately during hot weather periods. Public health campaigns and IPV screening in healthcare settings should be expanded during hot weather periods. Addressing structural inequalities, such as economic disparities, education gaps, and gender-based alcohol abuse, is vital for long-term prevention.

## 6. Conclusions

Extreme heat exposure was found to increase violence against women in both indoor and outdoor settings and across various socioeconomic, cultural, and geographical contexts. With rising global temperatures, understanding the heat–violence connection is crucial, especially in regions vulnerable to climatic changes and with already high rates of violence against women. Place-based studies integrating lived experiences with reported violence data are key to devising contextual risk reduction measures and also for emergency services resource planning.

## Figures and Tables

**Table 1:** Inclusion/Exclusion criteria.

Inclusion Criteria	Exclusion Criteria
No geographical limits or limits to publication dates English language peer-reviewed journal articles For quantitative studies, exposure variables included any temperature-related variables or indices derived from local weather observatories, or those linked to a specific hot weather event Data reported against violence or crime against women Qualitative studies including the lived experiences or general perceptions of female participants across the globe	Studies not related to violence or crime against women Review articles, commentaries, or perspectives without primary or secondary data analysis Studies solely exploring the seasonal variation in crime/violence data Qualitative studies that do not include female participants

**Table 2:** Data extraction table.

References	Climate Zone and Location	Population Characteristics	Temperature Contexts	Design/Methodology	Groups at Increased Risk	Findings
(Cohn, 1993) [19]	Continental climate United States	Victims of rape and domestic violence reported to police during the years 1985, 1987 and 1988	Daily average temperature ranged from—32 °C to 39 °C	Quantitative approach, forward multiple linear regression analysis of call data from Minneapolis Police Department (1985, 1987, 1988)	Women in domestic settings	Higher ambient temperatures, especially above 25 °C, are positively correlated with an increase in domestic violence incidents. Domestic violence is more likely to occur in the evening and at night, while rape incidents peak at night and on weekends.
(Henke & Hsu, 2020) [22]	Subtropical and continental climate United States (263 counties)	Women in intimate relationships, including various racial demographics and socioeconomic statuses	Average daily maximum temperature of 18.47 °C	Quantitative research method: the study utilized police report data from the National Incident-Based Reporting System (NIBRS) alongside county-level gender wage data, temperature reports, and demographic controls	Women in low-income households, women in interracial relationships where significant income disparities exist	Hot weather is associated with increased IPV, with each 1 °C rise linked to approximately a 1% increase in cases. An increase in women’s relative wages can reduce the risks.
(Nguyen, 2024) [15]	Tropical, semi-arid, subtropical, and highland climate 34 developing countries (Burkina Faso, Benin, Congo, Cote d’Ivoire, Cameroon, Dominican Republic, Egypt, Gabon, Ghana, Guatemala, Honduras, Haiti, India, Jordan, Kenya, Cambodia, Kyrgyz Rep., Mali, Myanmar, Malawi, Mozambique, Nigeria, Namibia, Nepal, Peru, Pakistan, Sierra Leone, Chad, Togo, Tajikistan, Tanzania, Uganda, Zambia, Zimbabwe	Women aged 15 to 49 years in rural and urban areas	Average temperature across the regions: 23.28 °C (standard deviation 5.936)	Quantitative analysis using Demographic and Health Surveys (DHS) and Climatic Research Unit Time Series (CRUTS) data from 2000 to 2018	Rural women, women from poor households, low-educated women, women with low-educated partners	An increase of 5.94 °C elevates the risk of any form of IPV by 3.7%.
(Otrachshenko et al., 2021) [18]	Subarctic, continental climate 79 regions in Russia	Women and men in Russia from 1989 to 2015: focus on violent mortality, with an analysis of gender and age differences	Extreme hot temperatures exceeding 25 degrees Celsius and cold temperatures falling below –23 degrees Celsius	Panel data analysis using regional-level data from 1989 to 2015	Women, especially those in domestic settings and regions with high unemployment and alcohol consumption rates	The findings show a gendered impact, with women more vulnerable to violence on hot days, particularly on weekends and in regions with alcohol abuse and unemployment.
(Rotton & Cohn, 2001) [20]	Continental climate United States	Domestic violence victims as reported in police call reports	Temperature range: 15 °C to 35 °C, with analysis of heat waves and seasonal variations	Analysis of police service calls in 1987 and 1988, examining the relationship between temperature and rates of domestic violence	Women in domestic settings	Domestic violence incidents were correlated with higher temperatures (over 30 °C).
(Sanz-Barbero et al., 2018) [17]	Mediterranean climate Spain	Women involved in intimate partner femicides (IPF), reports of IPV and 016 IPV telephone help line calls in the Community of Madrid from 05/01 to 09/30 in the years 2008–2016	During the months of May through September of 2008–2016, the average maximum temperature during the heat waves was 35.8 °C in Madrid	Ecological, longitudinal time series study	Married women	The risk of Intimate partner femicide increased three days after the heatwave, [RR(IC95%):1.40(1.00–1.97)]. Police reports of IPV increased one day after the heatwave [RR (IC95%):1.02(1.00–1.03)]. Help line calls increased five days after the heatwave [RR(IC95%):1.01(1.00–1.03)].
(Stevens et al., 2024) [16]	Subtropical climate Australia	Reported crimes of victims of violent crimes, including domestic violence, non-domestic violence, and sexual assaults	The average daily mean maximum temperature over the study period was 23.49 °C	Negative binomial regression models were applied to assess the relationship between temperature, humidity, and different types of violent crimes (13 years of data 2006 to 2018)	Victims of domestic violence, particularly in indoor settings, people living in low socioeconomic status (SES) areas, especially those without access to air conditioners	Domestic violence increased with rising temperatures, particularly in indoor settings
(Xu et al., 2021) [21]	Temperate, subtropical, continental climate United States (7 large Cities)	Cases of sex offenses (sodomy, fondling and rape) from 2007–2017	The average daily mean temperature for all observed days in the seven cities was 15.4 ± 9.2 °C, varying from 11.0 ± 11.1 °C in Chicago to 21.3 ± 7.8 °C in Tucson.	Time-stratified case-crossover design from 2007–2017	Women (90% of victims) and victims of sodomy, fondling and rape	An increase in daily mean temperature of 5 °C was associated with a 4.5% increase in sex offenses over the following 0–8 days. Increased risks were linked to offenses occurring in open spaces, educational institutions, and streets rather than residences
(Zhu et al., 2023) [14]	Tropical, sub-tropical, arid, temperate climate India, Nepal, Pakistan (South Asia/LMICs)	women aged 15 to 49 years	The annual temperature ranges were mostly between 20 °C and 30 °C	Cross-sectional study which used data from the Demographic and Health Survey (194,871 ever-partnered women aged 15 to 49 years)	Women from rural areas, lower household incomes, partially built homes as well as women with younger partners or partners with low educational background or lower income were considered to be at risk	An increase in IPV prevalence with higher annual mean temperature was found, with a 1 °C increase in the annual mean temperature associated with a 4.5% increase in IPV prevalence

## Data Availability

Not applicable.
